# The Effect of Face-to-Face Education and Educational Booklet on Heart Health Indexes of the Hospitalized Patients with Myocardial Infarction

**DOI:** 10.1155/2013/675634

**Published:** 2013-05-27

**Authors:** Safar Ali Esmaeili Vardanjani, Laleh Fanisaberi, Firozeh Alirezaee Shahraki, Ahmad Khalilzadeh, Abdolazim Tavakoli Vardanjani, Fatemeh Ghani Dehkordi

**Affiliations:** ^1^Shahrekord University of Medical Science, Shahrekord, Iran; ^2^Faculty of Nursing and Midwifery, Mazandaran University of Medical Science, Mazandaran, Iran; ^3^Ayatollah Kashani Hospital, Shahrekord University of Medical Science, Shahrekord, Iran; ^4^Tehran University of Medical Science, Tehran, Iran; ^5^Bushehr University of medical sciences, Bushehr, Iran

## Abstract

Management of risk factors and heart health indexes in the patients who have been diagnosed with myocardial infarction will result in prevention of secondary myocardial infarction, reduction of postimprovement mortality, increase of life span and improvement of life quality. Patient education has been found to be one of the most fundamental and essential care programs on the basis of identification and control of the patients' health criteria. The study is a quasi-experimental research consisting of two groups. In this study, 112 patients with myocardial infarction who were below the age of 70 were selected randomly and divided into two groups (case group and control group) after being matched based on age and sex. The researcher first measured the health indexes including smoking, cholesterol level, body mass, level of anxiety, and amount of systolic and diastolic blood pressure in patients who have been diagnosed with myocardial infarction for the first time. He performed education program in case group and analyzed the said variables after four months. He also compared the behaviors in the two groups after being educated. The data was analyzed by SPSS software, version 15 (This product is licensed to FeFDBi, ABiComputer, 1337), and the two groups were compared by using appropriate statistical tests. According to the results, after education period, systolic blood pressure of the case group improves compared with control group (*P* < 0.05/*P* = 0.022), case group tends to quit smoking more than control group does (*P* = 0.013), cholesterol level of case group improves compared with control group (*P* < 0.0001), changes of body mass are more positive in case group compared with control group (*P* = 0.012), and anxiety of case group reduces compared with control group (*P* < 0.0001).

## 1. Introduction

The heart is the most active organ of the body and takes a key role in the health and function of other organs, so that any failure in its function causes disorder and damage in other organs. Heart coronary artery diseases are the major cause of mortality in most of the developed countries [1] as well as developing countries and can affect all aspects of physical, mental, and social health [2].

In USA, cardiovascular disease is one of the most prevalent diseases causing hospitalization of adult people [3]. In this country, about 1.5 million myocardial infarctions occur per year. The mortality caused by acute myocardial infarction is around 30%. It has been estimated that one patient out of each 25 patients suffering from this disease dies during the first year [4].

In Iran, there is not so much statistics with respect to the prevalence of cardiovascular diseases and the mortality caused by it. However, due to increase of urbanization and mechanization of life, reduction of physical activity and increase of people's weight, increase in consumption of narcotics, occupational and mental stresses, and lack of attention to health recommendations, there is an increasing rate of heart diseases and the mortality caused by it, particularly in the city of Tehran [5]. According to WHO report of March 2002, 22% of mortality in the world and 35% of mortality in Iran are caused by cardiovascular diseases [1].

Among cardiovascular diseases, heart ischemic diseases are the main cause of mortality, disability, infliction of heavy financial losses, and waste of a great deal of capital throughout the world [1, 5]. Myocardial infarction is an acute, unpredictable disease causing physical and social limitations which entails rearrangement of the patient's life. This causes an increased anxiety in the patient [4, 6]. Heart failure creates a severe anxiety not only in the patient but also in his family [7]. If the patient survives in the first attack, subsequent problems may occur and the probability of the next killing attack increases [8]. From another point of view, most of such patients are between the age of 35 and 65 and are in fact considered the active arm of a society. The mortality or disability of these workforces may be an irreparable loss in the third-world countries which are facing the challenge of insufficient active workforces [9].

The main objective behind the management of risk factors in the patients with coronary arteries diseases is to reduce the risk of more coronary attacks and other arteriosclerotic risks, increase life span, and improve life quality, [10] which are performed in the form of secondary prevention. In clinical studies, the effect of several criteria as secondary prevention has been established. Medical criteria include treatment by aspirin, blood pressure control, and fat control. Life style criteria include increase of physical activity, use of healthier food regimes, and quitting smoking [11]. Therefore, the patients with heart failure are required to change health-related behaviors in their life. Fortunately, most of risk factors which cause coronary artery disease can be eliminated by personal health-related behaviors [12]. 

Nowadays, patient education is one of the most fundamental and essential care programs in medical treatment systems and is regarded as underlying part of the tasks and duties in all health professions [13]. Reduction of anxiety and the associated behaviors entails identification of lack of skills and a program for teaching special skills [14].

The goal behind patient education is to improve heart health status through promotion of health-related behaviors and involvement of the patient in care program and decision making in care period, reduce cardiovascular diseases, improve life quality, and reduce hospital costs [15].

In view of performed studies which indicate the reduction of stress by patient education and since few actions have been taken with respect to patient education after myocardial infarction, the researcher intends to carry out a study of random clinical trial type in order to investigate the effect of information on the amount of stress in patients with myocardial infarction and find out by research findings that whether the information given to patients has any effect in reduction of their stress.

Since the lack of educational program and regular rehabilitation in Iran for heart patients after being discharged from hospital and lack of the groundwork for activity of nurses, it is recommended that educational programs should be started in hospitals by nurses and be continued while establishing efficient relation.

It is hoped that this research can help in the enhancement of patient care quality and reduction of patients' anxiety by providing them with appropriate and sufficient information and necessary education.

## 2. Methods

The present research is of random clinical trial type. The researcher first measured health-related indexes including smoking, cholesterol level, body mass, anxiety level, and systolic and diastolic blood pressure in the patients who had been diagnosed with myocardial infarction for the first time. He executed education in case group and then investigated each of the said variables four months after education. He also compared these behaviors in case and control groups before and after education. Statistical population of the research consists of women and men below the age of 70 who had been diagnosed with myocardial infarction for the first time, were not suffering anxiety and psychosis-related diseases, were not medical and nursing staff, were able to read and write, had at least 40% ejection fraction after acute step of myocardial infarction, had been hospitalized in the selected hospitals of Iran University of Medical Sciences and Shahid Beheshti Medical University due to myocardial infarction, and were willing to take part in the research. Also, those who traveled abroad for treatment (e.g., surgery operation) during the said four months were omitted. In this study, the researcher attended the research location (Post-CCU, C.C.U, and Internal Heart Ward of the hospitals in question) every day in three shifts (morning, evening, and night) and took sample from the qualified persons. First, the case group with intended number of samples was selected and then the control group was selected from the same hospitals based on personal characteristics of the members of case group. The two groups were matched in terms of age and sex, and the samples were analyzed in a meaningful statistical level with 95% confidence and 80% test power and *μ*_1_ − *μ*_2_ = 5; *S* = 9.6 with prediction of 10% sample drop. The following formula was used:

(1) $$\begin{array}{c} N=\frac{1}{1-f}\times \frac{2{\left(Z_{\alpha }+Z_{\beta }\right)}^{2}S^{2}}{{\left(\mu_{1}-\mu_{2}\right)}^{2}}. \end{array}$$

And a total number of 120 patients with myocardial infarction in control and case groups was estimated. 

In this research, data was collected by two separate questionnaires. The first questionnaire consisted of two parts: the first part comprised demographic data including age, sex, education level, job, residence place, marriage status, income status, family history, and hospitalization records; the second part included short questions regarding the variables of body mass, smoking, level of cholesterol, and amount of systolic and diastolic blood pressure. Data concerning cholesterol level was extracted from the patients' case, and the questions regarding smoking and demographic data were directly asked and recorded in questionnaire. Systolic and diastolic blood pressure, weight, and height were measured by the researcher via a measurement tool. The second questionnaire contained 21 test questions each having four choices, with a point range from 0 to 3 and the maximum point was 63. Based on the points obtained, the patients were placed in one of these four groups: without anxiety (below 10 points), low anxiety (10–18 points), average anxiety (19–29 points), and high anxiety (30–63 points). This questionnaire was completed by the patients themselves. Scientific validity of the questionnaires was assessed by content validity.

The reliability of the questionnaires of personal data and questions concerning research variables showed Cronbach's alpha with 91% correlation coefficient. Scientific reliability of standard tools was assessed by Ms. Tahereh Ganji in the year 2005 in the study of the relation between anxiety and religious beliefs in nursing students of Nursing and Midwifery University of Iran by using reexamination whose correlation coefficient was 0.85. Therefore, the questionnaire was confirmed.

Patients were educated on symptoms, causes, and problems of myocardial infarction and change of life style such as quitting smoking, increase of physical activity, observance of food and drug regime, control of blood pressure, weight, and management of anxiety. When education was completed, the variables of blood pressure, level of anxiety, cholesterol level, body mass, and smoking were investigated at the end of the third month and then these indexes were compared with preeducation period. Education was performed in the form of individual education by researcher in the patient's room for 20 minutes and group education (2–5 patients) in hospital's clinic for 20 minutes by the method of face-to-face education and educational aid tools such as educational booklet given to patients. Followup was made continuously for a period of four months following the first visit by phone or face-to-face visit in order to review the topics and answer their questions. The patients were asked to attend the hospital for visit. Also a fixed telephone number and a mobile phone number were provided to answer their questions from 18:00 p.m. to 24:00 p.m. Then, at the end of the fourth month, the questionnaire concerning cholesterol level, body mass, systolic and diastolic blood pressure, smoking, and anxiety was completed and analyzed.

## 3. Results

The samples under study were 112 heart patients, out of which 50 persons (44.6%) were in control group and 62 persons (55.4%) were in case group. In terms of sex, 90 patients (80.4%) were male and 22 patients (19.6%) were female. As demonstrated by different research findings, heart diseases are more prevalent in males and this affected the number of male patients in the research. With respect to education level, 42% of the patients under study were in the level of primary school studies, 10.7% were in the level of middle school studies, 24.1% were in the level of high school studies, and 23.2% had academic studies. With respect to income, 11.6% of the patients under study were in a good economic status, 67.9% were in average economic status, and 20.5% were in bad economic status. In terms of marriage status, 93.8% of the patients were married and 6.2% were nonmarried (widow, divorced, or single). It is noteworthy that 75.9% of the patients did not have family records of myocardial infarction and 24.1% had family records of myocardial infarction. 46.3% of the patients had records of smoking and 53.7% had no records of smoking. Average age of the samples under study was about 55. 67.7% of the patients were 60 years of age or less. 85.5% of the patients were residing in Tehran. 50% of the patients in both groups stated that they have family records of myocardial infarction. 41.9% in case group and 32% in control group expressed background disease. Background diseases included Asthma, second type diabetes, hyperthyroid, and rheumatism. Diabetes had the highest frequency in the two groups, being 27.4% in the case group and 28% in the control group.

The results of statistical analysis by using *t*-test indicate that there is a 6.15-point difference in anxiety status between the two groups, which denotes that educational interventions have reduced anxiety average of the case group by 6.15 points. This difference is significant in the level 0.01.

In view of the results of the present research, Asilioglo and Celik (2004, Turkey) made a study on the effect of presurgery education on anxiety of open surgery patients. The results of this research indicated that clear and hidden anxiety of patients in case control was less than that of control group, but the difference was not meaningful. The researcher believes that these results may be due to failure in measurement of pre-surgery anxiety level of the patients and failure in matching the two groups. Among other reasons we can mention the lack of relation between care takers, clinical conditions of patients' room, and their relation with patients who had undergone surgery, performing education by researcher only between 8:00 and 17:00, and lack of education in postsurgery and postdischarge periods [16]. Also, Ms. Tahereh Dehdari et al. carried out a study on heart patients to determine the effect of a designed educational program on the reduction of patients' anxiety after coronary artery bypass surgery. The results of this research indicated that designing an education program based on precede-proceed pattern and self-efficiency theory can reduce the anxiety of patients after bypass surgery and consequently improve their life quality [14]. Furthermore, many other studies, such as the research made by Tahereh Ganji on the effect of education on the amount of knowledge and anxiety of patients hospitalized in Shahid Modarres Hospital of Tehran waiting for heart catheterization, denote a meaningful reduction of anxiety, which is in agreement with the present research [3].

The findings of analysis indicated that there is a meaningful difference in the level of 0.05 in systolic blood pressure between the two groups, which shows that education has resulted in reduction of systolic blood pressure. In other words, if patients are educated even in a limited manner after myocardial infarction, such education (which can be generally presented by nurses) can have a meaningful effect on reduction of blood pressure. This relative reduction becomes valuable when it is considered in line with other medical interventions. Shin et al (2008) carried out a research to analyze the effects of community-based management program on the patients suffering from hypertension. The results indicated that education and advice on the consumption of alcohol and smoking have positive effects on control of blood pressure, change of life style, exercise, and treatment of patients suffering from hypertension. However, more interventions are needed for long-term effects [17]. In The Netherlands, Kosan et al. (2008) carried out a study to evaluate the effect of internet program on treatment of cardiovascular risk factors. The results indicated that most of risk factors such as blood pressure reduce after six months of intervention and education via Internet [18]. Another study performed by Lin et al. (2008) on the effectiveness of community-based group education on control of blood pressure in patients with diabetes mellitus indicated a meaningful reduction of blood pressure [19]. In USA, Tang et al. (2008) performed a study to assess the effect of an acoustic relaxation tool on reduction of blood pressure and modification of heartbeats. The results denoted positive effect of two-minute relaxation on prediction from hypertension. The results of these studies are in agreement with ours. Of course, more research is recommended [20].

As shown in Tables 3 and 4, after education period, cholesterol level of the case group is less than that of the control group by 46.13 units, which is considered a meaningful difference in the level 0.001. In other words, education given after cardiovascular accidents can help patients adhere to health regimes and reduce their cholesterol level.

The findings of this study indicated that if heart patients are educated after such events as myocardial infarction, such education may have a considerable effect on health-related behaviors such as the risk factor of high cholesterol level in these patients.

In Greece, a research was carried out in 2009 under title of application of a strategy for management of dyslipidemia in order to study the effectiveness of an efficient strategy in the improvement of artery risk factor management in the patients with dyslipidemia, with or without cardiovascular disease. The results of this study showed 47%–63% reduction of the risk of estimated cardiovascular diseases in initial prevention as well as considerable increase of patients' preparedness for secondary prevention and achievement of the goals of cardiovascular disease risk factors from the base 29% to 79% after a period of 6 months [21].

Brittle et al. (2008) indicated that education, followup, and consultation to the patients result in promotion of activity level, life quality, and mental status of the patients [22]. Also, Caldwell et al. (2005) emphasized in their experimental study the positive effect of education programs and telephone followup on the knowledge, self-care behaviors, and disease symptoms of the patients with heart insufficiency [23]. All the above-said studies denote the importance of education in the management of chronic diseases. In general, identification of risk factors such as cholesterol reduction is the first step in health improvement, health-related activities, and disease prevention. Modification and control of risk factors often entail the change of health-related behaviors.

According to research results (Table 1), before education program, there were 48.4% and 42% smokers, respectively, in the case and the control groups. After education program, this rate decreases to 11.3% in the case group and 28.6% in the control group (Table 2). This indicates that education can help the reduction of smoking and increase of quitting smoking. Increase of quitting in the control group denotes that the importance of heart diseases and the patients becoming sensitive to their disease as well as general information given to patients by physicians and case specialists such as nurses can also affect temporary quitting. Investigations indicate that such adherence considerably reduces, particularly at the end of the first year. Therefore, long-term heart rehabilitation programs will have good efficiency in preservation of health indexes, promotion of life quality, and reduction of risk factors. This study, like many other studies, indicates the importance of education after cardiovascular events and its great effect on the reduction of risk factors. This study shows us that even limited education can result in behavioral changes which are highly important in prediction of secondary cardiovascular events, reduction of risk factors predicting cardiovascular problems, and promotion of health-related behaviors in patients after occurrence of myocardial infarction.

Plus et al. (2008) made a research on the effects of a developed health rehabilitation program on the patients under treatment after myocardial infarction or coronary artery bypass. Between May 1999 and May 2002, 828 patients below the age of 70 with myocardial infarction or waiting for coronary artery bypass were filtered for participation in the research at Dendride Hospital of Stockholm, Sweden. 531 patients were found unqualified and 73 patients did not accept to participate. The remaining 224 patients were randomly divided into case group (111 patients) and control group (113 patients). All patients in the two groups, with 37 smokers in total, participated in smoking quitting program. The results of this study indicated positive changes in improvement indexes and risk factors, including quit smoking, and significant advantages in the case group compared with the control group. This was perhaps because the control group received a broad intervention in their normal program in this study. These researchers believe that in order to improve heart rehabilitation, more attention should be paid to development of treatments and activities which reduce mental and social stresses, because these factors are highly connected with coronary artery diseases. The results of this study confirm our findings as well [24].

The results of statistical analysis by using independent *t*-test indicate that there is a difference of 0.62 points between two groups in body mass index. This denotes that education has resulted in reduction of body mass index average of case group by 0.62 points, which is a significant difference in the level 0.01 (Table 5).

A study made by Idolory et al. (2009) in Italy indicated the statistical reduction of all cardiovascular risk factors as well as 10% reduction in body weight [25].

## 4. Discussion

In fact, the result of the current study like scientific texts related to a cardiac rehabilitation program showed that education has an impact on reducing the risk factors. The dynamics of change in patients through psychological training interventions is focused today on cardiac rehabilitation program that in addition to training, it considers the principles of behavior change in a time domain that leads to a long-term change in lifestyle. Like many other studies, this study shows the importance of education after cardiovascular events in reducing risk factors and value of educational interventions in this field. This study showed us that even with limited trainings we will have behavioral changes that could be particularly valuable in the prevention of subsequent cardiovascular events, reducing the predictive risk factors of cardiovascular problems, and improving the health-related behaviors in patients after myocardial infarction. Today, cardiovascular diseases are considered as behavioral disorders, and also sustained behavioral change requires systematic and long-term interventions. 

According to decades of research, cardiology nurses can play an important role in cardiovascular rehabilitation spheres such as the initial training with the patients, consulting with spouses and families of these patients, transferring the necessary information, and guidance that other specialists cannot do. This study has confirmed the importance of educational interventions in patients after myocardial infarction and cardiovascular role that nurses can have in this area. Cardiovascular diseases are the most important cause of death in most countries. 

 In many countries, including Iran, these kinds of diseases are the main cause of using long-term disease-related benefits such as insurance and premature disability. Consequently, these diseases are considered as the main causes of health care costs and social insurances. Moreover, it has recently become clear that the nine most common and potentially modifiable risk factors, that is, lipoproteins, smoking, psychosocial factors, high blood pressure, alcohol consumption, lack of exercise, diabetes, abdominal obesity, and low fruit and vegetable consumption, are considered as the main causes in 90% of the population, that is, at the risk of myocardial infarction. These risk factors apply for both men and women in all parts of the world. Thus to reduce the risk factors, comprehensive cardiac rehabilitation programs including exercise, counseling with patient, psychological and education, and support after an acute event of ischemia or coronary artery bypass should be provided to all patients. Because cardiovascular diseases allocate 46% of death causes to themselves in Iran, naturally, the number of people who are afflicted with myocardial infarction is growing. Numerous psychological problems such as anxiety can be created for these patients. Psychological problems have negative effects on the prognosis and recurrence of heart disease and increase mortality in afflicted persons. According to research findings, anxiety is higher in these patients and requires further studies. Considering patient's training at any stage of disease process is necessary and inevitable. Finally, the researcher hopes that the authorities and medical practitioners in the country, according to the results of study, apply appropriate policies to improve cares after suffering from myocardial infarction that training is considered as its integral part. As being able to read and write was one of the criteria to participate in the study, the illiterate individuals were excluded from the study; it is suggested that a similar study should be carried out to evaluate the effect of training on heart health status measures in patients with myocardial infarction (by using manuals with most cartoon images of guiding signals) and comparing it with illiterate individuals to determine the appropriate training aid device for these persons. In this study, training was done regardless of personality factors, the way of learning, and the willingness of people to use the devices. So it is recommended that in doing a similar study, selection of the training aid devices should be based on the tendency of people and the results should be examined. By considering that in this research it was impossible to measure the awareness level of persons of health status standards before and after the intervention, and no study had been done in this field, also we did not have any questionnaire to assess these cases, it should be told that these are the limitations of our study.

## Figures and Tables

**Table 1:** Determination and comparison of level of anxiety, body mass index, cholesterol, blood pressure, and quitting smoking before education in the patients with myocardial infarction in the case and the control groups.

Specifications	Case group	Control group	Test results	Explanations
Body mass index (%)				Underweight ≤ 18.5
Normal	38.7	34	*P* value = 0.039 χ^2^ = 6.494	Normal weight = 18.5–24.9
Extra weight	43.5	62		Overweight = 25–29.9
Fat	17.7	4		Obesity = BMI of 30 or greater
Smoking (%)				
Yes	48.4	42	*P* value = 0.500	
No	51.6	58	χ^2^ = 0.455	
Cholesterol level (%)				
Normal	35.5	52	*P* value = 0.079	Normal ≤ 200
Abnormal	64.5	48	χ^2^ = 3.089	Abnormal = greater than 200
Systolic blood pressure (%)				
Normal	80.6	82	*P* value = 0.855	Normal ≤ 140 mm Hg
Abnormal	19.4	18	χ^2^ = 0.033	Abnormal = greater than 140 mm Hg
Systolic blood pressure ($\bar{X}\pm \text{SD}$)	126.8 ± 18.42	125 ± 19.92	*T* = 0.489 *P* value = 0.626	
Diastolic blood pressure (%)				
Normal	100	96	χ^2^ = 2.525	Normal ≤ 90 mm Hg
Abnormal	0	4	*P* value = 0.197	Abnormal = greater than 90 mm Hg
Diastolic blood pressure ($\bar{X}\pm \text{SD}$)	74.5 ± 9.175	74.8 ± 12.37	*T* = 1.113 *P* value = 0.266	
Anxiety status (%)				
No anxiety	25.8	36		
Low anxiety	43.6	34	χ^2^ = 3.618	
Average anxiety	12.9	20	*P* value = 0.306	
High anxiety	17.7	10		

**Table 2:** Determination and comparison of level of anxiety, body mass index, cholesterol, blood pressure, and quitting smoking four months after education in the patients with myocardial infarction in the case and the control groups.

Specifications	Case group	Control group	Test results
Body mass index (%)			
Normal	38.7	40	*P* value = 0.676 χ^2^ = 0.782
Extra weight	53.2	56	
Fat	8.1	4	
Smoking (%)			
Yes	11.3	30	*P value = 0.013
No	88.7	70	χ^2^ = 9.138
Cholesterol level (%)			
Normal	87.1	36	*P value ≤ 0.0001
Abnormal	12.9	64	χ^2^ = 3.476
Systolic blood pressure (%)			
Normal	95.2	76	*P value = 0.003
Abnormal	4.8	24	χ^2^ = 8.761
Systolic blood pressure ($\bar{X}\pm \text{SD}$)	125 ± 12.73	131 ± 19.67	*T* = 1.501 *P* value = 0.137
Diastolic blood pressure (%)			
Normal	98.4	90	χ^2^ = 0.087
Abnormal	1.6	10	*P* value = 3.840
Diastolic blood pressure ($\bar{X}\pm \text{SD}$)	124 ± 17.08	131 ± 19.67	*T* = 1.644 *P* value = 0.103
Anxiety status (%)			
No anxiety	48.4	30	
Low anxiety	32.3	34	χ^2^ = 0.127
Average anxiety	16.1	26	*P* value = 5.700
High anxiety	3.2	10	

*The obtained *P* is significant.

**Table 3:** Comparison of average and criterion deviation of anxiety level, body mass index, cholesterol level, and blood pressure in the patients with infarction before and after intervention in the control group 2011.

Control group/variables	Before intervention		After intervention		Statistical test
	Mean	SD	Mean	SD	
Cholesterol level	209.76	55.25	220.52	43.27	*t* = 1.839, *P*-value = 0.072
Systolic blood pressure	125	19.92	130.8	19.67	*t* = −2.66, *P-value= 0.010
Diastolic blood pressure	74.8	12.37	78.4	12.01	*t* = − 2.177, *P*-value= 0.034
Body mass index	26.19	3.65	25.91	3.54	*t* = 1.866,*P*-value = 0.068
Anxiety level	15.26	10.07	16.06	8.95	*t* = 1.273, *P*-value = 0.209

*The obtained *P* is significant.

**Table 4:** Comparison of average and criterion deviation of anxiety level, body mass index, cholesterol level, and blood pressure in the patients with myocardial infarction before and after intervention in the case group in 2011.

Case group/variables	Before intervention		After intervention		Statistical test
	Mean	SD	Mean	SD	
Cholesterol level	220.10	55.55	184.06	31.20	*t* = 5.839, *P-value < 0.001
Systolic blood pressure	126.77	18.42	125.97	12.73	*t* = 0.436, *P*-value = 0.664
Diastolic blood pressure	74.59	9.23	75.08	9.24	*t* = 0.455, *P*-value = 0.651
Body mass index	26.73	3.46	25.84	2.48	*t* = 4.544, *P-value < 0.001
Anxiety level	17.31	11.03	11.95	7.6	*t* = 7.446, *P-value < 0.001

*The obtained *P* is significant.

**Table 5:** Comparison of average and criterion deviation of changes in cholesterol level, systolic and diastolic blood pressure, body mass index, and anxiety level in the case and the control group after intervention in 2011.

Variables	Case group		Control group		Statistical test
	Mean	SD	Mean	SD	
Changes of cholesterol level	−36.03	48.59	+10.76	41.38	*P-value < 0.001, *t* = 2.408
Changes of systolic blood pressure	−0.8	14.54	+5.8	15.39	*P -value = 0.022, *t* = 2.328
Changes of diastolic blood pressure	0.49	8.45	3.6	11.69	*P*-value = 0.1191, *t* = 1.573
Changes of body mass index	−0.89	1.55	−0.27	1.03	*P-value = 0.012, *t* = 2.547
Changes of anxiety level	−5.35	5.66	0.8	4.44	*P-value < 0.001, *t* = 6.280

*The obtained *P* is significant.
